# Burning mouth syndrome: a review on diagnosis and treatment

**Published:** 2014

**Authors:** EC Coculescu, A Radu, BI Coculescu

**Affiliations:** * Department of Oral Medicine, Faculty of Dental Medicine, “Carol Davila” University of Medicine and Pharmacy, Bucharest; ** "Carol Davila” University of Medicine and Pharmacy, Bucharest, Academy of Economic Studies, Bucharest; *** Discipline of Microbiology, Faculty of Medicine, “Titu Maiorescu” University, Bucharest

**Keywords:** burning mouth syndrome, orofacial pain, diagnosis, treatment

## Abstract

Burning mouth syndrome (BMS) is defined as a chronic pain condition characterized by a burning sensation in the clinically healthy oral mucosa. It is difficult to diagnose BMS because there is a discrepancy between the severity, extensive objective pain felt by the patient and the absence of any clinical changes of the oral mucosa. This review presents some aspects of BMS, including its clinical diagnosis, classification, differential diagnosis, general treatment, evolution and prognosis.

## Introduction

Many studies of burning mouth syndrome (BMS) have described more epidemiological and etiological aspects than diagnosis and treatment [**1**]. This study analyzes the BMS symptoms and the presence of concomitant depressive disorders, mania, anxiety associated with this clinical entity. The data of this review were materialized in a standard examination protocol which included a clinical examination of the oral cavity, salivary flow rate and general hematology investigations, gastroenterology control (for type 3 BMS) and a psychiatric assessment for all the patients with BMS symptoms who were addressed to the clinical service of Oral Pathology, Faculty of Dental Medicine, “Carol Davila” University of Medicine and Pharmacy, Bucharest. All BMS cases were grouped into three clinical groups (**Table 1**). Also, many treatments with variable success were reviewed in this article.

**Clinical diagnosis**

The clinical history was helpful in diagnosing BMS [**2**]. Burning sensation in the oral mucosa syndrome was most often cited by patients but BMS might manifest as an itching sensation, numbness, taste alteration (the BMS patients reported ageusia for bitter/acid/spicy substances or metallic taste), dry mouth, burning pain, oral stinging, etc. These symptoms were almost always located in the tongue or oral mucous membranes, in more than one oral site, with the anterior two thirds of the tongue, the anterior hard palate and the mucosa of the lower lip being most frequently involved [**3**-**6**]. This does not mean that all the oral mucosa could be involved without the identification of any precise anatomical distribution. Once in place, disorders can be maintained for long periods of time, from several months to several years [**6**].

**Classification and subtypes**

The intensity and duration of symptoms can vary from patient to patient, this observation making some authors propose a classification of BMS in three clinical subtypes (**Table 1**) [**6**].

**Table 1:** Clinical forms of BMS [**7**,**8**]

**Type**	**Relative frequency**	**Symptoms**
1	35%	Present every day, but not at the wake. Occurence during the day and deepening in the evening, when intensity was the highest
2	55%	Present every day from the awakening.
3	10%	Present only a few days and located in unusual regions (neck).

Type 1 BMS was associated with systemic diseases such as nutritional deficiencies, diabetes mellitus, etc., type 2 was usually associated with psychological disorders, and type 3 BMS was related to allergic reactions or local factors [**7**,**8**].

The usefulness of this classification would be primarily related to the possibility of correlating the diagnosis with patient prognosis. It seemed that patients suffering from type 2 were most refractory to any kind of treatment [**6**,**9**].

The main symptoms were present in patients with BMS [**8**]:

a) The presence of the triad consisted of:

1. Pain in the oral mucosa: burning, scalding, tingling, numb feeling, swelling, stinging;

2. Altered taste (dysgeusia): persistence of a certain taste/ altered taste perception;

3. Xerostomia, with dry mouth.

b) Other associated symptoms: thirst, headache, pain in the temporomandibular joint (TMJ) tenderness/ pain in the masticatory and neck, shoulder, and suprahyoid muscles.

Scala et al. (2003) [**8**,**10**] proposed a set of positive diagnostic criteria for the identification of BMS difference between the fundamental criteria and additional criteria (**Table 2**).

**Table 2:** Criteria developed by Scala for the diagnosis of BMS [**8**,**10**]

**Fundamental criteria**	1. Daily deep burning sensation of oral mucosa (bilateral)
	2. Pain of at least 4-6 months
	3. Constant intensity or increasing intensity during the day
	4. Characteristic symptoms are not getting worse/ sometimes there may be an improvement over the ingestion of food and liquid
	5. No interference with sleep
**Additional criteria**	6. The occurrence of other oral symptoms (dysgeusia +/- xerostomia)
	7. Sensory changes/ chemosensory alterations
	8. Psychopathological alterations/ mood changes that translate the patient’s personality disorder

**Differential diagnosis**

BMS diagnosis was essentially one of exclusion [**11**,**12**]. It was based on a very thorough history and clinical examination. Often, the local clinical examination does not reveal any changes. Sometimes physical examination can detect minor changes or normal variations such as: cracked tongue, exfoliative glossitis of various origins, geographic tongue or white/ coated tongue [**2**,**6**,**13**].

If the physical examination revealed no clinically evident lesions in the oral mucosa, it was reasonable to suspect that intraoral burning was a possible indicator of systemic disorders (such as diabetes mellitus or anemia presence of blood with different etiologies: iron, folic acid, or vitamin B12 - cobalamin - etc.) [**6**].

**Table 3:** Diagnostic tests useful in the diagnosis of BMS

**Common laboratory tests [**6**]**	- Complete blood cell counts (CBC)
	- Sedimentation rate (ESR)
	- Serum iron
	- Serum ferritin concentration
	- Iron binding capacity
	- The concentration of circulating folic acid, vit. B12, zinc, etc.
	- Glycemia (blood glucose level)
	- Determination of serum hormone (estradiol) levels in women
**Other laboratory/ clinical tests**	- Sialometry
	- Specific investigations of systemic diseases
	- Allergic epicutaneous tests
	- Fungal culture for the isolation of Candida species from oral mucosa

The determination of the values of such parameters was a prerequisite for all the patients with oral algae, presenting clinically normal oral mucosa [**6**].

The other laboratory tests investigated serum antibodies against Helicobacter pylori and in Sjögren’s syndrome. Of the microbiological and fungal examinations, the presence of Candida albicans investigation was required in the oral cavity [**2**].

In most cases, patients with burns of the mouth and normal buccal mucosa showed normal biological constants. The identification results of the laboratory tests of a systemic disease (diabetes mellitus, iron deficiency, anemia etc.) required the establishment of its therapy, which will result in the mouth algae non-specific symptoms evanescence [**6**].

Sometimes, patch tests for contact allergy to dental materials such as zinc, cobalt, mercury, gold, palladium or food allergens as ascorbic acid, cinnamon, nicotinic acid, propylene glycol and benzoic acid revealed a diagnosis of burning mouth syndrome (BMS) [**1**,**14**-**17**].

**Treatment and Medical Management**

Since the treatment is generally unsatisfactory and BMS is a chronic pain syndrome, it is necessary that patients are properly informed regarding the expectations that need to be realistic, appropriate.

The first step in the treatment of BMS was subject to the differentiation of primary from secondary form because in the presence of the latter, therapy was directed to treating the causal disease. This etiologically directed therapy usually produces a good response [**18**]. Thus, in the presence of allergic contact reactions, the simple removal of the suspected allergen (e.g. the material/ dental alloy) determined the remission of the symptoms of BMS.

In the case of idiopathic BMS, the therapeutic principles coverd a triple purpose: improvement of symptoms, correction of biological and/ or morphological disturbances and the therapy of psychoemotional changes (**Table 4**) [**6**].

**Table 4:** The major therapies used in BMS [**6**]

**Symptomatic therapy**	**Correction therapy**	**Psychopharmacological therapy**
Solution 3% benzydamine hydrochloride	Iron	Benzodiazepines
Antihistamines	Vit. B12 / folate	Tricyclic antidepressants (TCAs)
Sucralfate	Vit. B1, B2, B6	Monoamine oxidase inhibitors (MAOIs)
Lidocaine	Estrogen therapy	Serotonergic antidepressants
Capsaicin	Neuroleptics	Antipsychotics
Salivary substitutes	Topical antifungal	Hypnosis

Therapeutic strategies included benzodiazepines (clonazepam), tricyclic antidepressants (amitriptyline), anticonvulsants (gabapentin), selective inhibitors of serotonin receptors (paroxetine and sertraline), capsaicin topical/ systemic, alpha-lipoic acid (neurological antioxidant), benzydamine hydrochloride at 0,15% or 3%, hormone replacement therapy, vitamins supplementation and/ or zinc, iron and psihocognitiva therapy [**6**].

As an adjunctive therapy method, acupuncture is referred to in the art as being beneficial for the relief of symptoms in patients with BMS [**19**].

It was necessary to integrate the different pathogenic mechanisms which were hypothetically incriminated in determining the intended therapy. The multifactorial origin of BMS hypothesis suggested a therapeutic intervention aimed at correcting any changes, local or systemic, individualized for each patient based on laboratory results [**1**,**6**].

Psychological hypothesis aimed at controlling and mitigating the psychical disturbances occurred. The products used, solution of benzydamine HCl, sucralfate, and antihistamines, lidocaine, capsaicin, have not proved statistically significant improvements: in most patients subjective manifestations remained unchanged [**6**,**9**,**20**].

However, the current level of knowledge about the disease does not have any certainly effective treatment. The treatment conduct of BMS included the counseling process, possibly applied by a physician who demonstrated empathy for the patient [**6**]. The purpose of counseling was to provide patient information and explanations about the sickness, about benign lesion notions of correlation with the field (age and sex). These patients should always know that their disease is most often related to stress and if they let it go, at least in part, the state of pain may reduce [**6**].

**Evolution and prognosis of BMS**

In an 18 months retroprospective study, Sardella et al. (2006) showed that 28,3% of the cases proved a moderate improvement and 49% had no significant change, and, in 18,9% of the cases there was a worsening of the symptoms in patients who have not received any treatment. The complete spontaneous remission is rare and has been reported by the same team in 3% of the cases investigated for a period of 5 years [**21**,**22**].

## Conclusion

It can be stated that in patients with BMS, psychiatric examination is always needed. The above disorders can be overlooked by a dental exam and their treatment will be in collaboration with a psychologist or a psychiatrist. About 50% of the patients presented psychiatric disorders such as anxiety, depression, obsessive or psychosomatic symptoms. This incidence was significantly higher than the incidence of these disorders in the normal population (8-16%). However, it is equally possible that patients with chronic pain acquire these disorders later [**12**].

Nevertheless, typically, the prevalence of BMS dramatically increases with age [**1**,**15**]. In all cases, modern interdisciplinary approach is needed to solve the diagnostic dilemmas of BMS [**2**,**4**].
